# Unique Plant Resources and Distribution Patterns in the Valley Forest of the Irtysh River Basin

**DOI:** 10.3390/plants13141957

**Published:** 2024-07-17

**Authors:** Ling Xu, Tong Liu, Zhifang Xue, Jihu Song, Ye Yuan, Zidong Zhang, Yongyu Chen

**Affiliations:** 1 College of Life Science, Shihezi University, Shihezi 832003, China; xl9602@126.com (L.X.); xuezhifang@stu.shzu.edu.cn (Z.X.); songjihu@stu.shzu.edu.cn (J.S.); yuanye@stu.shzu.edu.cn (Y.Y.); zhangzidong@stu.shzu.edu.cn (Z.Z.); chenyongyu@stu.shzu.edu.cn (Y.C.); 2 Xinjiang Production and Construction Corps Key Laboratory of Oasis Town and Mountain-Basin System Ecology, Shihezi 832003, China

**Keywords:** valley forest, flora, resource value, Irtysh River Basin, distribution, life form, medicinal plant

## Abstract

The river valley forests of the Irtysh River Basin are a germplasm bank of *Salicaceae* species and rare plant resources in China, and the distribution varies with the river and is highly distinctive. However, there is a dearth of systematic research on the characteristics of plant resources. In this study, a comprehensive investigation was conducted in the trunk stream and six tributaries with valley forest distribution in the Irtysh River Basin, and 244 quadrats were set up. The analysis focused on the composition of the flora and resource characteristics. The results reveal the following: (1) The valley forests of the Irtysh River Basin contain 256 species of plants belonging to 57 families and 178 genera, among which 19 species of trees, 23 species of shrubs, and 214 species of herbs were investigated. (2) Among the identified species, 226 (88.67%) were recognized as resource plants, with medicinal plants being the most abundant (176 species, 68.75% of the total). (3) The distribution patterns of trees, shrubs, and herbs of each resource type vary across rivers. Elevation drop, river length, and river distance all significantly affect the number of specie. This study elucidated the current status and distributional characteristics of plant resources in the valley forests of the Irtysh River Basin, which is essential for both biodiversity conservation and sustainable resource utilization.

## 1. Introduction

The valley forests are forests developed on river floodplains along rivers or streams, usually growing in lowland river valleys within the range of periodic floods [1] and are an important part of forest resources in arid and semi-arid regions. They are important not only for nitrogen storage and distribution [2], carbon sinks and water conservation [3], and mitigation of global climate change [4] but also for the maintenance of biodiversity [5], the integrity of ecosystems [6,7], the security of water and agricultural resources, and local agricultural and livestock production [8].

The valley forests of the Irtysh River Basin have unique plant species and vegetation types, dominated by plants of the *Salicaceae* and *Betulaceae* species [9], and are the concentrated distribution area of many species of *Salicaceae* in the world as well as the biodiversity hotspot area of *Salicaceae* [10]. *Populus alba*, *Populus nigra*, and many other *poplar* species are naturally distributed in the same drainage basin and are unique; *Populus canescens* distribution patterns show diversity with geography [10]. The watershed area within China is 59,000 km^2^, with a river length of 633 km [11]. The trunk stream and six main tributaries are the main distribution areas of river valley forests [12], and the maximum altitude difference is as high as 2240 m, with remarkable spatial heterogeneity of habitats [11]. The unique topographic and geomorphological features have nurtured many rare and endemic plant resources and rich germplasm resources [13,14].

However, flora and tree–shrub–herb resource surveys of the tributaries and trunk stream have not received comprehensive attention in these biodiversity-rich valley forests. In particular, disturbances resulting from anthropogenic activities such as hydraulic engineering [15], land-use alteration [16], and grazing [17] have significantly increased vulnerability, which leads to the depletion of forest resources, a continuous reduction in woodland ecosystem coverage [18], and a decline in the value of ecosystem services with substantial spatial heterogeneity in gains and losses [19], as well as the endangerment of numerous plant species. Consequently, there is an urgent need for systematic studies on flora and resources.

Previous studies on the forest flora of the river valleys in the Irtysh River Basin were mainly focused on the localization of specific geographic areas. Studies have conducted analyses on the floral composition in various locations, including the Pre-Altai plain valley forests [20], Altay prefecture [21], the Xiaodonggou forest area of Altay Mountains, the Beitun forest area [22], and the Koksu Wetlands in Xinjiang. Regrettably, research on the flora composition of valley forests within river basins has predominantly focused on a single river and a specific life form [23,24,25]. Although scholar have proposed recommendations for the conservation of poplar plant resources in the Irtysh River Basin [9], there is a lack of comprehensive comparative research on the floristic characteristics of valley forests and the current status of plant resource development and utilization within the Irtysh River Basin.

The diverse range of plant resource values fulfills essential human needs, such as healthcare, livestock, and food, which are irreplaceable by technology [26,27,28,29]. This significance is particularly pronounced in local communities and developing nations [30,31]. Understanding the distribution patterns of the species used by people is thus essential for the sustainable management of plant resources [32]. Although the large geographical distribution of terrestrial plants has been extensively investigated globally [33], and the utilization, conservation, and management of plant resources in various regions across the globe have already garnered significant attention [31,34], the primary regulatory challenge lies in the dearth of local research data [35]. Therefore, in the context of the Irtysh River Basin’s valley forests, it is imperative to elucidate the categorization and distribution of plant resources as a fundamental prerequisite for effective conservation and sustainable exploitation. Consequently, comprehensive and systematic data support assumes utmost significance.

In light of this, a comprehensive survey was conducted on the distribution of valley forests in both plains (low elevation areas with relatively flat topography) and mountains (gully areas in low to mid-mountainous zones) along the six main tributaries and trunk stream within the Irtysh River Basin, with a view to clarifying (1) the composition characteristics of wild vascular flora in valley forests within the basin; (2) the types of plant resource values in valley forests; (3) the family, genus, and life form characteristics of valley forests resource plants; (4) differences in the distribution of wild vascular and resource plants among tributaries and the trunk stream; and (5) key plant species. In this manner, the present status of plant resources in the valley forests will be investigated, elucidating their potential value and providing comprehensive and systematic data support for inventorying the background resources of valley forests in the Irtysh River Basin. This will serve to effectively protect biodiversity in the valley forests and facilitate future rational development and utilization of these resources.

## 2. Results

### 2.1. Characterizing the Composition of Wild Vascular Plant Flora in Valley Forests

#### 2.1.1. Characterization of the Composition of the Quantitative Structure at the Species Level within the Family

The wild vascular flora in the Irtysh River Basin encompasses a total of 256 species, distributed among 57 families and 178 genera. Among these, there are 19 tree species, 23 shrub species, and 214 herbaceous species (Table A1).

According to the number of species within each family [36] and considering the composition characteristics of wild vascular plant families in the Irtysh River Basin, we classified the 57 plant families in this region into four distinct types: monospecific families (with 1 species), few families (with 2–5 species), multispecies families (with 6–10 species), larger families (>10 species) (Table 1). The five larger families, *Asteraceae*, *Gramineae*, *Fabaceae*, *Rosaceae*, and *Salicaceae*, constitute only 8.77% of the total number of families; however, they account for 123 species or 48.05% of the total number of species (nearly half of all species), making them the main component in the quantitative structure at the species level. The monospecific families comprise 28 families, which account for 49.12% of the total number of families, thus exerting a dominant influence on the composition of plant families.

#### 2.1.2. Characterization of the Composition of the Quantitative Structure at the Species Level within the Genus

According to the number of species within each genus [36] and considering the composition characteristics of wild vascular plant genera in the Irtysh River Basin, we classified the 178 plant genera in this region into three distinct types: monospecific genera (with 1 species), few genera (with 2–5 species), multispecies genera (>5 species) (Table 1). The multispecies genera comprise only two genera of *Populus* and *Salix*, encompassing a total of 15 species, which accounts for 8.43% of the total number of species and holds significant importance. Monospecific genera, comprising 133 genera, account for 74.72% of the total number of genera and represent 51.95% of the total number of species, indicating their predominant presence and relatively rich diversity of genera.

### 2.2. Types of Plant Resources and Species Composition of Valley Forests

Among the wild vascular plants of the valley forests in the Irtysh River Basin, a total of 18 families, 64 genera, and 85 species of forage plants were identified (Table A1), constituting 31.58%, 35.96%, and 33.2% of the respective total numbers of families, genera, and species. *Poaceae* (20 species in 19 genera), *Fabaceae* (20 species in 8 genera), and *Asteraceae* (13 species in 10 genera) accounted for more than half of the total species, which constituted the main body of forage plants in this area.

Medicinal plants encompass a total of 176 species belonging to 51 families and 139 genera (Table A1), constituting 89.47%, 78.09%, and 68.75% of the respective total number of families, genera, and species. *Asteraceae* (19 genera and 29 species), *Rosaceae* (13 genera and 16 species), *Fabaceae* (10 genera and 16 species), and *Poaceae* (13 genera and 13 species) exhibit dominance in the area. The area possesses abundant medicinal plant resources at the levels of family, genus, and species.

Edible plants encompass a total of 75 species belonging to 29 families and 64 genera (Table A1), constituting 50.88%, 35.96%, and 29.30% of the respective total number of families, genera, and species. Edible plants are more distributed in *Asteraceae* (9 genera and 12 species) and *Rosaceae* (9 genera and 10 species).

Economic plants encompass a total of 71 species belonging to 28 families and 56 genera (Table A1), constituting 49.12%, 31.46%, and 27.73% of the respective total number of families, genera, and species. The families with more economic plants are *Salicaceae* (2 genera and 13 species), *Asteraceae* (7 genera and 7 species), *Rosaceae* (9 genera and 10 species), and *Poaceae* (8 genera and 9 species).

Ornamental plants encompass a total of 94 species belonging to 38 families and 73 genera (Table A1), constituting 66.67%, 41.01%, and 36.72% of the respective total number of families, genera, and species. Quantitative structure exhibits a distinct dominance at the family level, exemplified by *Asteraceae* (9 genera and 9 species), *Rosaceae* (7 genera and 11 species), and *Fabaceae* (6 genera and 12 species).

Cultural plants encompass a total of 30 species belonging to 19 families and 28 genera (Table A1), constituting 33.33%, 15.73%, and 11.72% of the respective total number of families, genera, and species. These include *Asteraceae* (6 genera and 6 species), *Fabaceae* (4 genera and 4 species), and *Rosaceae* (2 genera and 3 species), as well as *Salicaceae*, *Plantaginaceae*, *Lamiaceae*, *Cannabaceae*, *Poaceae*, *Malvaceae, Orchidaceae*, *Polygonaceae*, *Geraniaceae*, *Ranunculaceae*, *Cyperaceae, Amaryllidaceae*, *Caryophyllaceae*, *Amaranthaceae*, *Linaceae*, and *Boraginaceae*, each with 1 genus and 1 species.

### 2.3. Family, Genus and Life Form Characteristics of Resource Plants in Valley Forest

#### 2.3.1. Distribution of Resource Plants in the Family and Genus

The valley forests of the Irtysh River Basin harbor a total of 256 species of wild vascular plants, among which resource plants account for a proportion of 87.11% (Table A1). Resource plants encompassed a total of 227 species, distributed across 54 families and 162 genera, constituting 94.74%, 91.01%, and 88.67% of the overall family, genus, and species in the region, respectively. *Asteraceae* species are clearly dominant in all types of resource plants. *Asteraceae* (22 genera and 33 species), *Rosaceae* (14 genera and 18 species), *Fabaceae* (10 genera and 24 species), *Poaceae* (21 genera and 26 species), and *Salicaceae* (2 genera and 13 species) are the dominant families of the resource plants. The number of genera and species contained in these five families account for 38.76% and 44.53% of the total number of genera and species in the region. The composition of resource plants in the study area predominantly exhibits a concentration within large families, thereby mirroring the family distribution patterns observed among wild vascular plants in the region.

#### 2.3.2. Life Form Characteristics of Resource Plants

**Figure 1:**
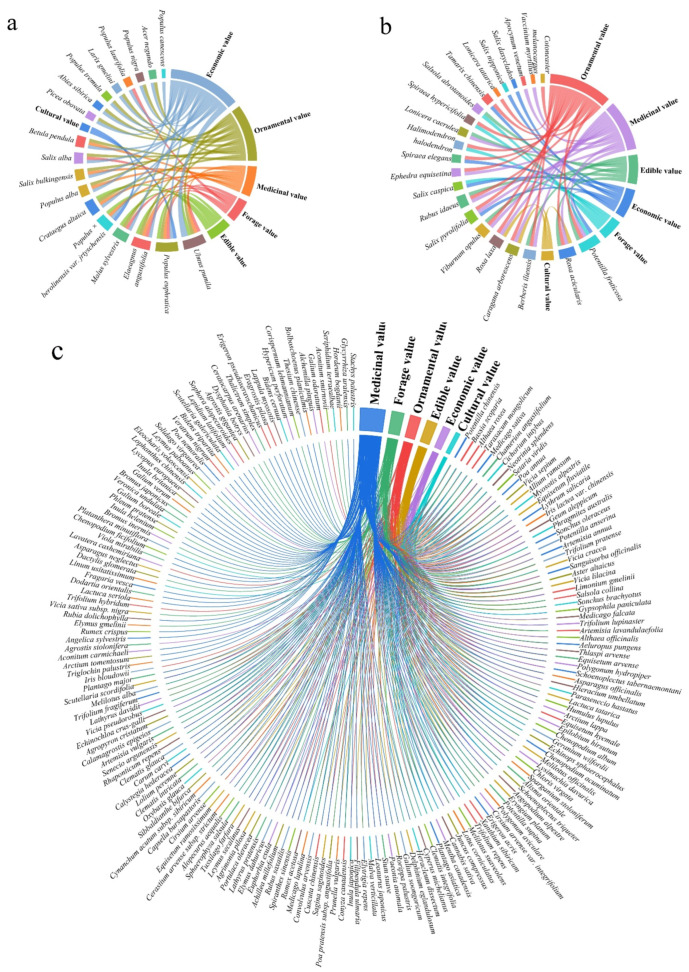
Resource plant composition among various life forms in the valley forests of the Irtysh River Basin: (**a**) 18 species of trees; (**b**) 22 species of shrubs; (**c**) 187 species of herbs.

The wild vascular plants of the valley forests in the Irtysh River Basin comprise 19 tree species, 23 shrub species, and 214 herbaceous species (Table A1). Notably (Figure 1), an impressive proportion of them possess significant resource value, with 94.74% of trees (18 species), 91.30% of shrubs (22 species), and 87.38% of herbs (187 species). Economic and ornamental plants in trees are highly represented under this life form, with 94.74% and 68.42%. Ornamental and medicinal plants in shrubs are highly represented under this life form, with 65.22% and 56.52%, respectively. Herbs have a high proportion of forage plants (34.58%) in addition to a prominent proportion of medicinal plants.

**Figure 2:**
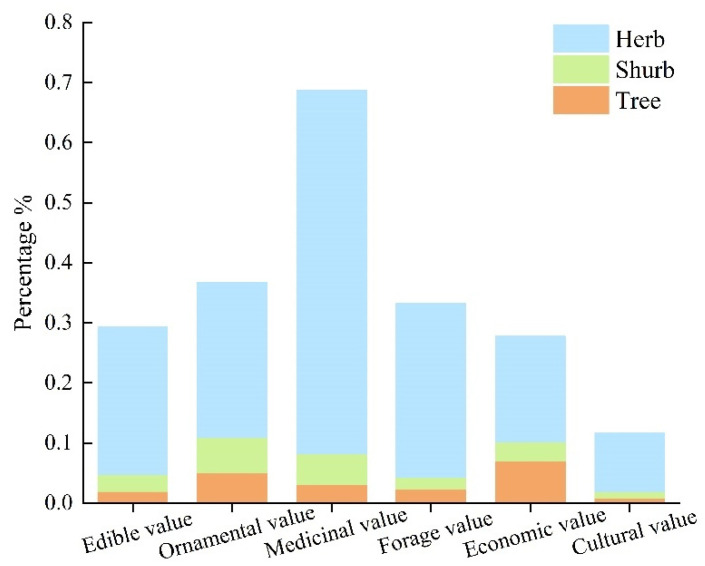
Percentage of life forms for each plant resource value type in the river valley forests of the Irtysh River Basin.

The percentage of life forms for each plant resource value type indicates that medicinal plant resources are the most abundant in this region (Figure 2). Regardless of the resource type, the number of herbaceous resource species accounts for a much higher percentage of the total wild vascular plant species in this area (9.77–60.55%) compared to tree resources (0.78–7.03%) and shrub resources (1.17–5.86%). This high number of herbaceous species greatly enriches the plant resources of this region.

### 2.4. Differences in Species Distributions among the Rivers of Valley Forests

#### 2.4.1. Differences in Distribution of Wild Vascular Plants in Tributaries and Trunk Stream

**Figure 3:**
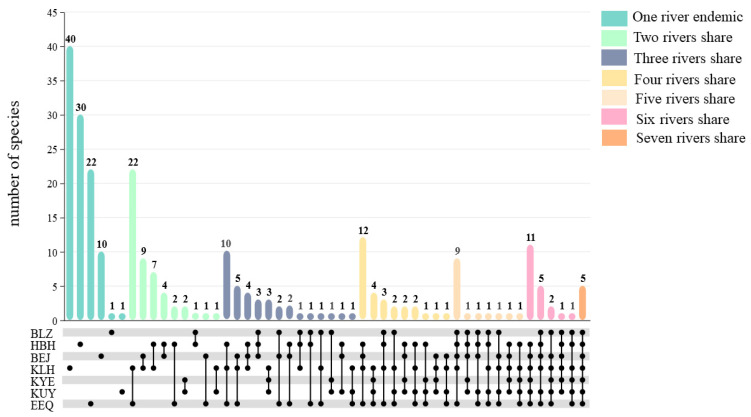
Distribution of endemic species among rivers in the valley forests of the Irtysh River Basin. BLZ: Berezek River; HBH: Haba River; BEJ: Burqin River; KLH: Crane River; KYE: Kayertes River; KUY: Kuilters River; EEQ: trunk stream. The lower part of the line represents the subgroups with shared species between the corresponding rivers. The upper part shows the number of unique and shared species in each subgroup.

The number of species varied between rivers, with 43 species in the Berezek River, 123 species in the Haba River, 94 species in the Burqin River, 167 species in the Crane River, 37 species in the Kayertes River, 54 species in the Kuilters River, and 131 species in the trunk stream (Table A1). Of these species, 104 occur in only one river, representing 42.63% of the total number of species, meaning that close to half of the species are endemic to one river. As shown in Figure 3, the Crane River had the most endemic species at 23.95% (40 species), followed by the Haba River at 24.39% (30 species), the trunk stream at 16.79% (22 species), and the Burqin River at 10.64% (10 species). One species that occurs only in the Berezek River is *Salix cinerea*, one species that occurs only in the Kuilters River is *Chloris virgata*, and there are no endemic species in the Kayertes River.

**Figure 4:**
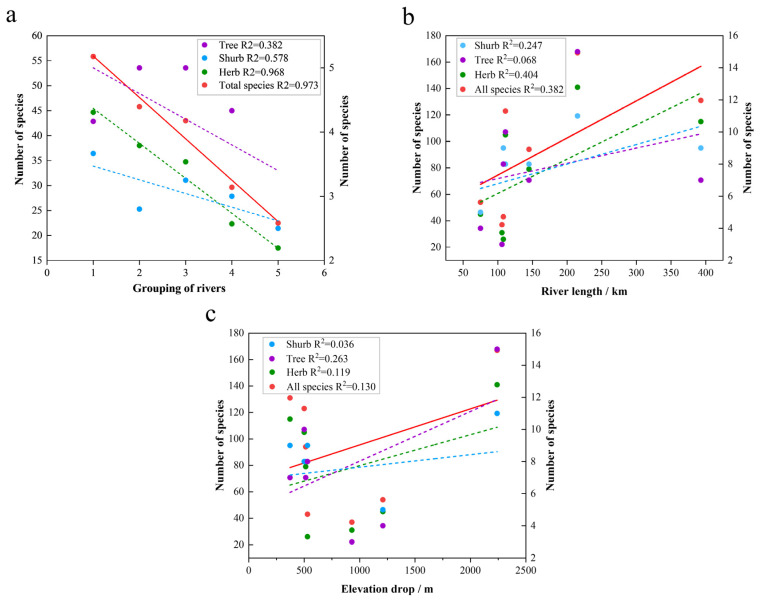
(**a**) Variation in the number of shared species of wild vascular plants with distance between two rivers. (**b**) Variation in the number of species of wild vascular plants of different life forms with river length; (**c**) Variation in the number of species of wild vascular plants of different life forms with elevation drop. The left *y*-axis shows the number of total species and species of herbs, and the right *y*-axis shows the number of species of shrubs and trees. In the *x*-axis of figure (**a**), 1 represents the grouping of tributaries with the main stream, 2 represents the grouping of adjacent tributaries, 3 represents the grouping of one tributary at an interval, 4 represents the grouping of two tributaries at an interval, and 5 represents the grouping of three tributaries at an interval. The Irtysh River Basin is a prototypical comb-shaped hydrological system, with the greater number of rivers spaced apart by tributaries indicating the greater distance between the two rivers.

As shown in Table A2, it is evident that the distribution of species shows a trend of having a large number of species in rivers with a high shared species between rivers. The highest number of species were shared between the Crane River and the trunk stream, with 22 species occurring only in these two rivers and none found in the other rivers. In addition, the subgroup with the most shared species was always associated with the trunk stream (Figure 3), i.e., the shared species between tributaries and the trunk stream were always higher than the shared species between tributaries, regardless of how many streams were compared (Figure 4a). Moreover, shared species are higher between geographically adjacent tributaries than between non-adjacent tributaries (Figure 4a).

#### 2.4.2. Characterization of Resource Plant Distribution in Tributaries and Trunk Stream

**Figure 5:**
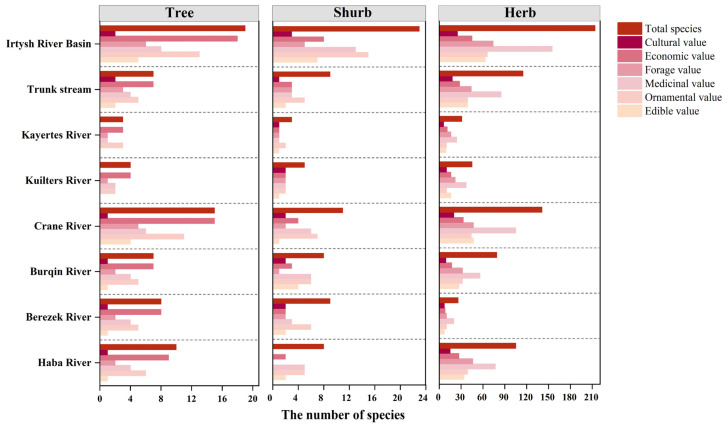
Types of plant resource values under different life forms in tributaries and trunk stream valley forests of the Irtysh River Basin.

The percentage of resource plants among the wild vascular plants in each river is more than 90%, especially in the Kuilters River, where 96.3% of the plants are resource plants (Figure 5). The Crane River had the highest number of resource species (152), which accounted for 66.96% of the resource plants in the whole basin, followed by the trunk stream with 52.86% (120 species), the Haba River with 50.22% (114 species), the Burqin River with 38.33% (87 species), the Berezek River with 18.06% (41 species), the Kayertes with 15.42% (35 species), and the Kuilters River with 22.91% (52 species).

The distribution patterns of resource plants vary across the rivers according to their life forms (Figure 5). Tree resources exhibit the highest economic value across all streams, followed by their ornamental significance. Shrubs have inconsistent distribution patterns in all rivers except the Haba and Burzin Rivers, which are identical. Herbaceous plants have slightly more food value than ornamental value in the Crane River and Kuilters River, and the rest of the resource values in each category have a similar distribution pattern across the rivers (Figure 5).

### 2.5. Key Plant Species

The results (Table 2) show that there were five species listed in the List of Wild Plants under State Key Protection (abbreviated as “State Protection”), five species listed in the List of Wild Plants under Xinjiang Protection, two species listed in the Convention on International Trade in Endangered Species of Wild Fauna and Flora (abbreviated as “CITES”), three species listed in the IUCN Red List of Threatened Species, six species listed in the Redlist of China’s Biodiversity (abbreviated as “RLCB”), two species listed in the China Species Red List (abbreviated as “CSRL”), and two species endemic to the area.

## 3. Discussion

### 3.1. Characteristics of Flora Composition of Valley Forests

The Irtysh River Basin exhibits a diverse array of valley forest vegetation. Although species are evidently relatively sparse compared to the Amazon lowland forest [37], the Indus River basin forest [38], and the Jinsha River dry–hot valley forest [39], the species richness in the riparian forests of the Irtysh River Basin (57 families, 178 genera, and 256 species) is remarkably high for the arid desert area of northwest China, especially when compared to the adjacent Junggar Basin desert area (30 families, 121 genera, and 245 species), which shares similar climate and close geographical conditions. The Altai pre-mountain plain through which the Irtysh River flows belongs to the northern edge of the Junggar Basin and has a temperate continental climate. The non-zonal vegetation in the valley forests, which relies on surface runoff, exhibits an aggregated distribution and is encompassed by zonal vegetation such as semi-deserts and deserts [20]. This contrasts with the distribution of desert vegetation is contingent upon natural precipitation patterns.

The distribution of flora within the families of valley forests exhibits an uneven pattern, with a concentration in a few larger families and dispersion towards monospecific families and genera. This distribution is closely associated with the regional origin of the flora, as well as climatic and historical factors. The region is situated in the arid area of northwest China, and its flora was formed during the Quaternary period. Its composition is primarily derived from the Angara flora, with a minor component from the ancient Mediterranean flora, and is dominated by temperate elements [36]. As a result, 48.05% of the species in this region are concentrated in five dominant families: *Asteraceae*, *Gramineae*, *Fabaceae*, *Rosaceae*, and *Salicaceae*. The dominant families are all cosmopolitan families, reflecting the region’s harsh climate and sensitive vegetation [40]; most plant species have difficulty surviving because of their narrow ecological range.

Emphasis should also be placed on those families that, although not dominant in terms of the number of species included, are typical families of the Irtysh River Basin. For instance, the Betulaceae family, represented by a single species, *Betula pendula*, has a high importance value and is a dominant species in Haba River and the Burqin River valley forests [41]. Similarly, there is only one genus and one species of *Ulmaceae* in Xinjiang, China, but it forms a dominant and even constructive layer in the vegetation of the mountain valleys [42]. Although the *Pinaceae* family has only three species, *Picea obovate* is the constructive species in montane valley forests [43]. Special attention should be given to these species in biodiversity conservation efforts.

### 3.2. Resource Values Vary by Life Form, with Medicinal Plants Being the Richest

The valley forests of the Irtysh River Basin are rich, with a total of 227 species accounting for 88.67% of the total number of species that can be directly utilized as resources. This is much higher than the flora of the forests of the western coast of Mexico (49.44%). Trees, as the highest biomass plants in valley forests, have great economic value. A remarkable 94.74% of these trees are resource plants, all of which have economic value (100%). For example, *Betula pendula*, *Abies sibirica*, *Picea obovata*, *Populus tremula*, *Populus alba*, *Populus canescens*, etc. can be extracted as industrial raw material. *Malus sieversii* is the ancestor of the world’s cultivated apples [44] and has become a potential advantage for local economic development [45]. *Crataegus altaica* is the main planting species for gardening and forestation [46]. *Populus euphratica* serves as a high-quality fuel source and *populus euphratica* resins have become an additional industry [47]. Herbaceous plant resources, although low in biomass share, are greatly enriched by their high species number for various types of river valley forests throughout the watershed, especially medicinal resources (Figure 3).

Similar to the results of the survey of plant resources in other regions of the world, the largest number of medicinal plant resources (68.75%) was found in the valley forests of the Irtysh River Basin. This result is similar or higher than those based on ethnobotanical surveys, such as 70% of medicinal plants in Ladakh in the Trans-Himalayan Region and 51.4% of medicinal plants in the Western Himalayan Region [48,49]. According to the statistics of the “List of medicinal plants in China”, medicinal plant resources in Changbaishan Nature Reserve accounted for 87.06% [50], which is higher than that of this study area, highlighting the need for enhanced conservation efforts in the Irtysh River Basin. The medicinal plant resources in this region are very rich at the level of family, genus, and species, with *Asteraceae* as the main family, and most of the life forms are herbs (60.55%). For example, *Salvia deserta Jacobaea argunensis*, *Dodartia orientalis*, etc., have the effect of clearing heat and detoxification. Meanwhile, endemic medicinal plant resources in Xinjiang, China, such as *Vaccinium myrtillus* and *Alchemilla pinguis* [51], are mainly distributed in mountain valley forests along the southern edge of the Altai Mountains, which are located at relatively high elevations. The Altai Mountains are one of the concentrated distribution areas of endemic species in Xinjiang [52], and the existence of mountains increases the region’s species richness [53,54].

River floodplains are habitats for endangered plants [10]. Many important resource plants, such as *Salix burqinensis*, *Abies sibirica*, *Populus euphratica*, *Populus euphratica*, and *Populus laurifolia*, along with herbaceous plants like *Berberis iliensis* and *Limonium gmelinii*, are already in an endangered state (Table 2). Although there has been recent improvement in vegetation cover [55], the direct damage caused by anthropogenic disturbances such as agricultural farming and grazing [16], along with the construction of water projects [56], poses a threat to these species, and conservation measures need to be continued to prevent their extinction. In addition, the valley forests are also distributed with many harmful plant such as *Echinochloa crus-galli* and *Polygonum aviculare*, which are the worst weeds in farmland and require attention [57].

### 3.3. Uneven Distribution of Species among Rivers

The number of species in tributaries and trunk streams ranges from 37 to 167, with an uneven distribution, as in the case of the Crane River, which has a maximum distribution of 167 species (Table A2). This can be attributed to several factors: Firstly, the length of the river (215 km) is greater than the other tributaries due to the fact that the longer the length of the river, the more species there are (Figure 4b). Secondly, it is favorable for species richness because the large elevation drop (2240 m) shapes a richer diversity of habitats (Figure 4c) [58]. Thirdly, the abundance of riverine forests in the Crane River, with the highest number of tree species (15), may provide sufficient resources for the shrub and herbaceous species and also favor species richness [59,60,61]. The Kayertes River, on the contrary, is short and located at the headwaters of the Irtysh River Basin, where species are scarce.

**Figure 6:**
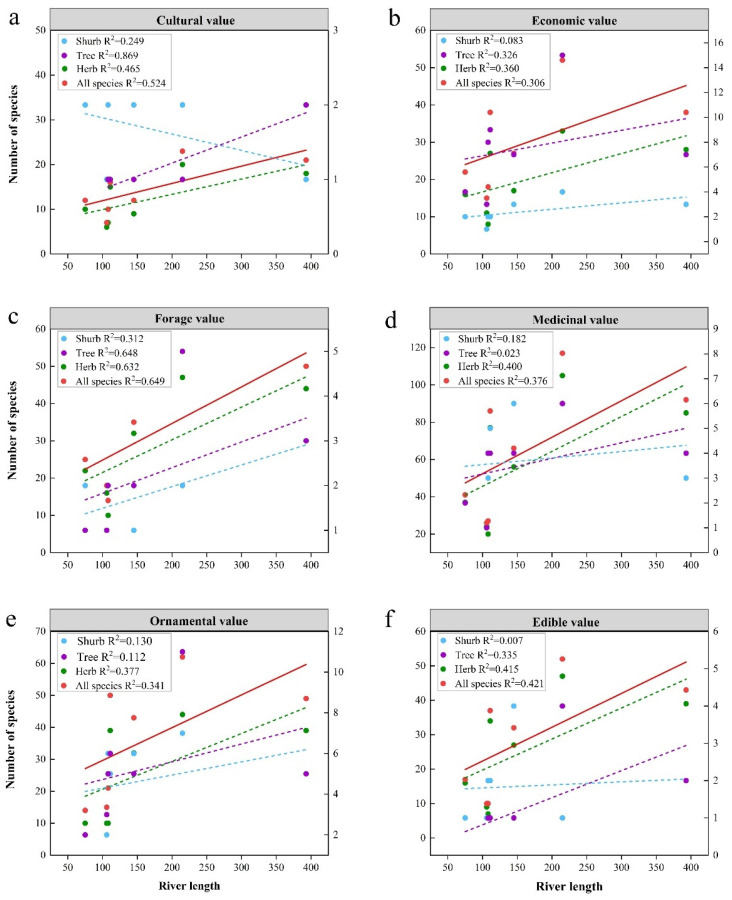
Variation in the number of plant species of each resource value type with river length: (**a**) cultural value; (**b**) economic value; (**c**) forage value; (**d**) medicinal value; (**e**) ornamental value; (**f**) edible value. The left *y*-axis shows the number of total species and species of herbs, and the right *y*-axis shows the number of species of shrubs and trees.

The number of species shared between tributaries and the trunk stream is generally higher than between different tributaries. This is likely due to the fact that the longer trunk stream has greater habitat diversity, and more frequent seed or propagule exchange with the tributaries via water flow [62,63]. The region is a prototypical comb-shaped hydrological system (Figure 6), where species shared between a tributary and its neighboring river are more numerous than those between non-adjacent rivers. This is significantly correlated with geographic distance—the closer the geographic distance, the more it facilitates seed dispersal and increases the number of shared species (Figure 4a). Across the entire Irtysh River Basin, only five species are found in all seven rivers. *Populus laurifolia* and *Thalictrum flavum* belong to the north temperate distribution type. The northern latitude of Xinjiang, China, is the main concentration of this distribution type. *Poa annua* is a cosmopolitan distribution type that dominates a wide range of habitats with its strong adaptive capacity. *Cannabis sativa* belongs to subtropical to the temperate distribution type as common species in Xinjiang. *Oxybasis glauca* belongs to the pantropical distribution type, which is undoubtedly a legacy of historical causes, reflecting the mild salinity that characterizes the soils of the valley forests of the Irtysh River Basin [36,64,65].

The different distribution patterns of plant resources with different life forms under each river reflect the fact that habitat heterogeneity is favorable for nurturing plant resources. River length significantly affects the number of species of each resource value type, especially herbaceous plants (Figure 6). Nearly half of the species (103 species) are endemic to a single river, with the majority (38.46%) occurring in the Crane River (Figure 3), including six species of trees (*Acer negundo*, *Elaeagnus angustifolia*, *Abies sibirica*, *Larix sibirica*, *Populus euphratica Ulmus pumila*), four species of shrubs (*Vaccinium myrtillus*, *Spiraea elegans*, *Caragana arborescens*, *Berberis iliensis*), and 30 species of herbs (Table A2). Most of them are found in montane valley forests, whose diverse habitats provide suitable habitat for these species.

## 4. Materials and Methods

### 4.1. Study Area

**Figure 7:**
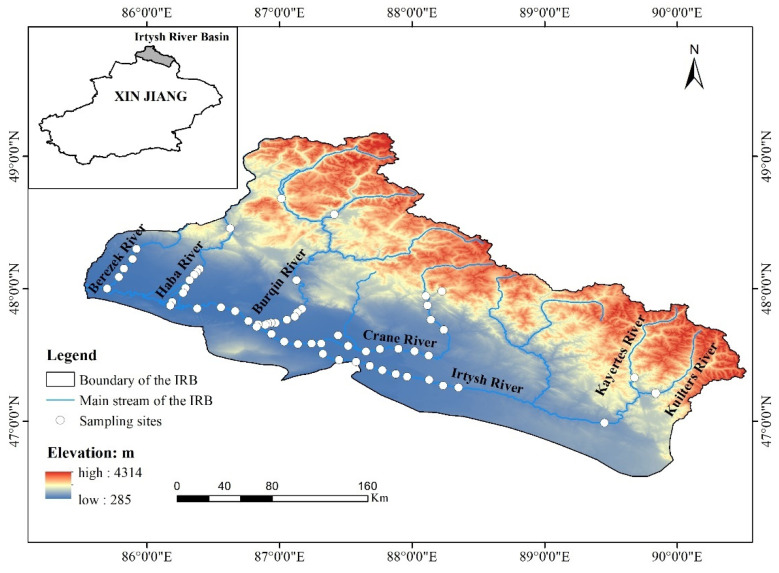
Overview map of the study area. Remarks: the stream section with no sample points are those with very little valley forests.

The Irtysh River Basin is situated in the Altay Prefecture of Xinjiang, China (85.51–91.06° E, 45.00–49.01° N), which occupies a central position within the Asian continent and experiences a temperate continental arid climate. The trunk stream originates from the southern foothills of the Altai Mountains, and the whole basin shows the terrain characteristics of high north and low south, high west and low east. The main tributaries and trunk stream make up a typical natural river valley forest distribution area and is the only area where poplar species are numerous and have a relatively concentrated natural distribution, in terms of plant distribution, and it belongs to the transition area between Central Asian flora and European flora [66]. The main tributaries with valley forests distribution encompass the Crane River (total length 215 km), the Burqin River (total length 145 km), the Haba River (total length 111 km), the Berezek River (total length 108 km), the Kayertes River (total length 106 km), and the Kuilters River (total length 75 km), and they are distributed on the northern side of the main channel and converge into the trunk stream to form the Irtysh River (total length 393 km) [11], thus constituting a prototypical comb-shaped hydrological system (Figure 7).

### 4.2. Field Investigations

Field surveys have been conducted along six main tributaries and the trunk stream of the Irtysh River Basin during the peak growing season of valley forests from June to September in 2022 and 2023. The habitat determines plant community structure [67,68]. We conducted a comprehensive reconnaissance survey of the overall distribution of valley forests, with a focus on identifying the different habitat types that arise with elevation gradients, as well as areas of dominant species distribution. Therefore, the distance between sample plots is not equal. For plain valley forests, sample plots were set up at 6–8 km intervals from the mouths of the tributaries. For montane valley forests, sample plots were set up at three elevation gradients: 900 m, 1100 m, and 1400 m. Among them, there are 5 sample plots in Bilizik River, 9 sample plots in Haba River, 13 sample plots in Burqin River, 13 sample plots in Crane River, 1 sample plot in Kayertes River, 1 sample plot in Kuilters River, and 19 sample plots in the trunk stream, with a total of 61 sample plots, of which 49 are in the plains and 12 are in the mountains.

Within each sample plot, quadrats were set up by the typical species focal sampling method, for a total of 244 tree quadrats, 30 m × 30 m, 488 shrub quadrats, 10 m × 10 m, and 1220 herbaceous quadrats, 1 m × 1 m. For the tree layer, there were four quadrats for each sample plot; each 30 m × 30 m tree layer quadrat was divided into nine 10 m × 10 m sub-quadrats via a contiguous grid quadrat method. For the shrub layer in the quadrat, due to the scarcity and uneven distribution of species, we investigated all the shrubs in each tree layer with a density of <5 plants, and if ≥5 plants, we set up two 10 m × 10 m shrub layer sub-quadrat along the diagonal. The center and corners of each quadrat were equipped with five herbaceous sub-samples measuring 1 m × 1 m. The species in the tree layer (with a diameter at breast height ≥ 3 cm), shrub layer, and herb layer were respectively recorded [41]. The field personnel responsible for plant identification have passed the Botany course examination, and for unidentified species in the sample plots, specimens were collected and subsequently examined by experts in the laboratory.

### 4.3. Data Processing

Plant identification and species information were accurately supplemented using references such as the “Flora of China”, “Concise Flora of Xinjiang”, iPlant Flora (https://www.iplant.cn/ (accessed on 2 May 2024)), Chinese Virtual Herbarium (CVH, https://www.cvh.ac.cn/ (accessed on 2 May 2024)), National Specimen Information Infrastructure (NSII), and the Chinese Plant Species Information Database (http://db.kib.ac.cn/eflora (accessed on 2 May 2024)). The information includes the family name, genus name, species name, and life form of each plant. According to the Information System of Chinese Rare and Endangered Plants (https://www.plantplus.cn/rep/ (accessed on 16 May 2024)), the National Register of Protected Species (http://protection.especies.cn/ (accessed on 2 May 2024)), and “List of Wild Plants under State Key Protection” (https://www.gov.cn/zhengce/zhengceku/2021-09/09/content_5636409.htm (accessed on 16 May 2024)), this study searched and evaluated the endangered species rank of the valley forests in the Irtysh River Basin. In light of the composition and characteristics of the resources within the Irtysh River Basin, referring to Pladias (https://pladias.cz/en/taxon/ (accessed on 20 May 2024)) and Leda (https://uol.de/en/landeco/research/leda/data-files (accessed on 20 May 2024)) databases, a comprehensive analysis and organization of the region’s floristic resources in the district was conducted. According to the classification system based on different utilization methods, plants are categorized into six resource types with direct value: medicinal, edible, forage, economic, cultural, and ornamental.

Origin statistical analysis was employed to depict the life form characteristics of plant resource types through the chord diagram and stacked bar chart. The bar chart facilitated the comparison of differences in the distribution of resource value types among rivers. The relationship between species difference and environmental factors was examined using linear fitting analysis. Additionally, an upset chart was generated via the Bioinformatics website to visually represent species differences between rivers by presenting information on pooled shared and endemic species.

## 5. Conclusions

(1) The Irtysh River Basin valley forests boast 256 species across 178 genera and 57 families and is rich in plant species in the arid region of northwest China. The flora composition is concentrated in *Asteraceae*, *Gramineae*, *Fabaceae*, *Rosaceae*, and *Salicaceae*. Of these species, 74.72% are monospecific genera, indicating a complex flora composition in the district. In terms of the number of species, Crane River > trunk stream > Haba River > Burqin River > Kayertes River > Kuilters River. The number of species increased significantly with increasing elevation drop and river length. The number of shared species decreased notably with increasing distance between rivers. (2) The Irtysh River Basin has 226 species of plants (88.67%) that can be utilized as resources, with the largest number of medicinal plant resources. Resource values are not consistent across life forms. Economically valuable plants were predominantly trees (100%), while ornamental species were more common among shrubs (65.22%) and medicinal plants among herbaceous species (72.43%). In addition, there are 19 species that require focused conservation and research. River valley forests are unique habitats that harbor diverse and rich plant resources, calling for protection and attention from stakeholders.

## Figures and Tables

**Table 1:** Quantitative composition of species within families and the genera of wild vascular plants in the valley forests of the Irtysh River Basin.

Number of Species in the Family	Number of Family	Percentage of Total Number of Family (%)	Number of Species in the Genus	Number of Genus	Percentage of Total Number of Genus (%)
1	28	49.12	1	133	74.72
2–5	16	28.07	2–5	43	24.16
6–10	8	14.04	>5	2	1.12
>10	5	8.77			
Total	57	100.00	Total	178	100.00

**Table 2:** Rare and endangered plant resources of the valley forests of the Irtysh River Basin.

Number	Species	State Protection	Xinjiang Protection	IUCN	CITES	RLCB	CSRL	Endemic Species
1	*Limonium gmelinii*					VU		
2	*Plantago maxima*					VU		
3	*Glycyrrhiza uralensis*	II	I					
4	*Inula helenium*							
5	*Platanthera minutiflora*	II			II			
6	*Ephedra equisetina*	II						
7	*Ranunculus altaicus*					NT		
8	*Malus sylvestris*	II	I	VU				
9	*Abies sibirica*		I	EN				
10	*Salix cinerea*					NT		
11	*Populus × berolinensis var. jrtyschensis*	II	II					√
12	*Populus laurifolia*					NT		
13	*Populus pilosa*					EN		
14	*Populus canescens*							√
15	*Apocynum venetum*		I					
16	*Salix burqingensis*						EN	
17	*Populus euphratica*						VU	
18	*Berberis iliensis*			VU				
19	*Spiranthes sinensis*	II			II			

NT: Near Threatened, VU: Vulnerable, EN: Endangered; Iand II indicate the level of protection, ‘√’ indicates an occurrence.

## Data Availability

All data are contained within the article.

## Appendix A

**Table A1:** Plant species composition and resource types of valley forests in the Irtysh River Basin.

Life Form (Number)	Family	Genus	Species	Edible Value	Ornamental Value	Medicinal Value	Forage Value	Economic Value	Cultural Value
Tree (19)	*Salicaceae*	*Salix*	*Salix alba*		√		√	√	
			*Salix bulkingensis*		√	√		√	
		*Populus*	*Populus × berolinensis var. jrtyschensis*	√		√		√	√
			*Populus nigra*		√			√	
			*Populus euphratica*	√	√	√	√	√	√
			*Populus laurifolia*				√	√	
			*Populus tremula*		√			√	
			*Populus pilosa*						
			*Populus alba*		√	√		√	
			*Populus canescens*					√	
	*Betulaceae*	*Betula*	*Betula pendula*		√	√		√	
	*Pinaceae*	*Abies*	*Abies sibirica*		√			√	
		*Larix*	*Larix gmelini*		√			√	
		*Picea*	*Picea obovata*		√			√	
	*Rosaceae*	*Malus*	*Malus sylvestris*	√	√		√	√	
		*Crataegus*	*Crataegus altaica*	√	√	√		√	
	*Ulmaceae*	*Ulmus*	*Ulmus pumila*	√	√	√	√	√	
	*Elaeagnaceae*	*Elaeagnus*	*Elaeagnus angustifolia*	√	√	√	√	√	
	*Sapindaceae*	*Acer*	*Acer negundo*		√			√	
Shrub (23)	*Salicaceae*	*Salix*	*Salix cinerea*						
			*Salix pyrolifolia*		√	√		√	
			*Salix dasyclados*					√	
			*Salix nipponica*					√	
			*Salix caspica*				√	√	
	*Rosaceae*	*Dasiphora*	*Potentilla fruticosa*	√	√	√	√	√	
		*Rosa*	*Rosa acicularis*	√	√	√			√
			*Rosa laxa*		√	√			√
		*Spiraea*	*Spiraea hypericifolia*		√		√		
			*Spiraea elegans*		√			√	
		*Rubus*	*Rubus idaeus*	√	√	√			
		*Cotoneaster*	*Cotoneaster melanocarpus*			√			
	*Fabaceae*	*Caragana*	*Halimodendron halodendron*		√		√		
			*Caragana arborescens*		√	√			√
	*Tamaricaceae*	*Tamarix*	*Tamarix chinensis*		√			√	
	*Vaccinium*	*Vaccinium*	*Vaccinium myrtillus*			√			
	*Apocynaceae*	*Apocynum*	*Apocynum venetum*			√			
	*Ephedraceae*	*Ephedra*	*Ephedra equisetina*		√	√			
	*Caprifoliaceae*	*Lonicera*	*Lonicera caerulea*	√		√			
			*Lonicera tatarica*		√				
	*Adoxaceae*	*Viburnum*	*Viburnum opulus*	√	√	√			
	*Amaranthaceae*	*Oreosalsola*	*Salsola abrotanoides*	√			√		
	*Berberidaceae*	*Berberis*	*Berberis iliensis*	√	√	√			
Herb (214)	*Fabaceae*	*Lotus*	*Lotus corniculatus*		√	√	√		
		*Melilotus*	*Melilotus alba*			√	√		
			*Melilotus suaveolens*			√	√	√	
			*Melilotus officinalis*			√	√	√	
		*Trifolium*	*Trifolium repens*			√	√		√
			*Trifolium fragiferum*		√		√		
			*Trifolium pratense*	√	√	√	√		
			*Trifolium lupinaster*			√	√	√	
			*Trifolium hybridum*		√				
		*Glycyrrhiza*	*Glycyrrhiza uralensis*			√			
		*Sophora*	*Sophora alopecuroides*			√			
		*Sphaerophysa*	*Sphaerophysa salsula*			√	√		
		*Medicago*	*Medicago sativa*	√	√	√	√		√
			*Medicago lupulina*		√		√		
			*Medicago falcata*	√			√	√	
		*Lathyrus*	*Lathyrus davidii*		√		√		
			*Lathyrus pratensis*			√	√		
		*Vicia*	*Vicia lilacina*	√	√	√	√		
			*Vicia pseudorobus*			√	√		
			*Vicia cracca*	√	√		√		√
			*Vicia sepium*	√	√	√	√		
			*Vicia sativa subsp. nigra*				√		
	*Poaceae*	*Echinochloa*	*Echinochloa crus-galli*			√		√	
		*Agropyron*	*Agropyron cristatum*			√	√		
		*Hordeum*	*Hordeum bogdanii*				√		
		*Roegneria*	*Elymus gmelinii*				√		
		*Calamagrostis*	*Calamagrostis epigeios*			√	√		
		*Setaria*	*Setaria viridis*	√	√	√	√	√	
		*Lolium*	*Lolium perenne*				√	√	
		*Chloris*	*Chloris virgata*	√		√	√		
		*Eragrostis*	*Eragrostis pilosa*			√			
			*Eragrostis minor*						
		*Neotrinia*	*Neotrinia splendens*	√		√	√	√	√
		*Agrostis*	*Agrostis gigantea*				√		
		*Agrostis*	*Agrostis stolonifera*					√	
		*Alopecurus*	*Alopecurus aequalis*			√	√		
		*Leymus*	*Leymus secalinus*		√	√			
			*Leymus paboanus*				√		
		*Phragmites*	*Phragmites australis*		√	√	√	√	
		*Elymus*	*Elymus dahuricus*				√	√	
		*Bromus*	*Bromus japonicus*			√			
			*Bromus inermis*				√		
		*Phleum*	*Phleum pratense*				√		
		*Dactylis*	*Dactylis glomerata*				√		
		*Elytrigia*	*Elytrigia repens*		√		√		
		*Poa*	*Poa nemoralis*					√	
			*Poa pratensis subsp. angustifolia*		√		√		
			*Poa annua*		√	√	√	√	
		*Aeluropus*	*Aeluropus micrantherus*						
			*Aeluropus pungens*		√	√	√		
	*Asteraceae*	*Xanthium*	*Xanthium sibiricum*			√		√	√
		*Carduus*	*Carduus nutans*						
		*Erigeron*	*Erigeron acris*		√	√			√
			*Erigeron pseudoseravschanicus*			√			
			*Conyza canadensis*			√	√		
		*Bidens*	*Bidens tripartita*			√			
			*Bidens cernua*			√			
		*Artemisia*	*Artemisia vulgaris*			√	√		
			*Artemisia annua*	√		√	√	√	
			*Artemisia lavandulaefolia*	√		√	√		
			*Artemisia scoparia*						
		*Cirsium*	*Cirsium arvense var. integrifolium*	√		√	√		
			*Cirsium arvense*	√	√				
		*Jacobaea*	*Senecio argunensis*		√	√			
		*Cichorium*	*Cichorium intybus*	√	√	√		√	√
		*Seriphidium*	*Seriphidium terraealbae*				√		
		*Sonchus*	*Sonchus oleraceus*	√		√	√		√
			*Sonchus transcaspicus*						
			*Sonchus brachyotus*	√		√	√		
		*Tussilago*	*Tussilago farfara*		√	√			
		*Echinops*	*Echinops sphaerocephalus*		√	√			√
		*Rhaponticum*	*Rhaponticum repens*			√	√		
		*Arctium*	*Arctium tomentosum*			√			
			*Arctium lappa*	√		√		√	
		*Tragopogon*	*Tragopogon orientalis*						
		*Taraxacum*	*Taraxacum mongolicum*	√	√	√		√	√
		*Hieracium*	*Hieracium umbellatum*			√	√	√	
		*Achillea*	*Achillea millefolium*		√	√			
		*Lactuca*	*Lactuca tatarica*	√		√	√		
			*Lactuca seriola*			√			
		*Parasenecio*	*Parasenecio hastatus*	√			√	√	
		*Inula*	*Inula britanica*			√			
			*Inula helenium*			√			
			*Inula japonica*		√	√			
		*Solidago*	*Solidago virgaurea*			√			
		*Aster*	*Aster altaicus*	√	√	√	√		
	*Ranunculaceae*	*Delphinium*	*Delphinium eglandulosum*		√				√
		*Ranunculus*	*Ranunculus altaicus*						
		*Thalictrum*	*Thalictrum flavum*						
			*Thalictrum simplex*			√			
		*Clematis*	*Clematis glauca*		√	√			
			*Clematis intricata*		√	√			
			*Clematis integrifolia*		√				√
		*Aconitum*	*Aconitum smirnovii*		√				
			*Aconitum carmichaeli*			√			
	*Equisetaceae*	*Equisetum*	*Equisetum ramosissimum*			√		√	
			*Equisetum hyemale*		√	√	√		
			*Equisetum fluviatile*	√		√	√	√	
			*Equisetum arvense*	√	√	√			
	*Rubiaceae*	*Galium*	*Galium boreale*			√			
			*Galium odoratum*					√	
			*Galium verum*			√			
			*Galium soongoricum*	√		√			
		*Rubia*	*Rubia rezniczenkoana*						
			*Rubia dolichophylla*			√			
	*Rosaceae*	*Fragaria*	*Fragaria vesca*			√			
		*Sanguisorba*	*Sanguisorba officinalis*	√	√	√		√	
		*Argentina*	*Potentilla anserina*	√		√	√	√	
		*Agrimonia*	*Agrimonia pilosa*			√		√	
		*Geum*	*Geum aleppicum*	√	√	√		√	
		*Sibbaldianthe*	*Sibbaldianthe bifurca*			√	√		
		*Potentilla*	*Potentilla supina*	√		√	√		
			*Potentilla desertorum*						
			*Potentilla chinensis*	√	√	√	√	√	√
		*Filipendula*	*Filipendula ulmaria*	√		√			
		*Rubus*	*Rubus saxatilis*		√	√			
		*Alchemilla*	*Alchemilla pinguis*				√		
	*Apiaceae*	*Vicatia*	*Vicatia atrosanguinea*						
		*Eryngium*	*Eryngium planum*	√	√	√			
		*Angelica*	*Angelica sylvestris*			√			
		*Heracleum*	*Heracleum dissectum*	√		√			
		*Carum*	*Carum carvi*	√		√			
		*Cenolophium*	*Cenolophium denudatum*						
		*Aegopodium*	*Aegopodium alpestre*	√		√		√	
		*Sium*	*Sium suave*		√	√			
	*Cyperaceae*	*Eleocharis*	*Eleocharis yokoscensis*			√			
		*Scirpus*	*Schoenoplectus triqueter*		√	√			√
			*Schoenoplectus tabernaemontani*		√	√		√	
		*Bolboschoenus*	*Bolboschoenus planiculmis*			√			
		*Cyperus*	*Cyperus fuscus*						
			*Cyperus michelianus*			√	√		
		*Carex*	*Carex altaica*						
	*Brassicaceae*	*Lepidium*	*Lepidium latifolium*			√			
		*Rorippa*	*Rorippa palustris*	√		√			
		*Capsella*	*Capsella bursapastoris*	√		√			
		*Cardaria*	*Lepidium chalepense*						
		*Meniocus*	*Alyssum linifolium*						
		*Thlaspi*	*Thlaspi arvense*	√		√		√	
	*Caryophyllaceae*	*Stellaria*	*Stellaria media*						
		*Cerastium*	*Cerastium arvense subsp. Strictum*	√		√			
			*Cerastium lithospermifolium*						
		*Sagina*	*Sagina saginoides*		√	√			
		*Gypsophila*	*Gypsophila paniculata*			√		√	√
		*Silene*	*Silene graminifolia*						
	*Amaranthaceae*	*Corispermum*	*Corispermum lehmannianum*				√		
		*Ceratocarpus*	*Ceratocarpus arenarius*				√		
		*Chenopodium*	*Chenopodium acuminatum*	√		√	√		
			*Chenopodium album*	√		√	√		
			*Chenopodium ficifolium*			√			
		*Bassia*	*Bassia scoparia*	√	√	√	√	√	√
		*Oxybasis*	*Oxybasis glauca*	√				√	
		*Dysphania*	*Dysphania botrys*				√		
		*Salsola*	*Salsola collina*	√		√	√		
	*Convolvulaceae*	*Calystegia*	*Calystegia hederacea*	√		√			
		*Cuscuta*	*Cuscuta chinensis*	√		√			
		*Convolvulus*	*Convolvulus arvensis*			√	√		
	*Linaceae*	*Linum*	*Linum usitatissimum*						√
	*Iridaceae*	*Iris*	*Iris lactea var. chinensis*		√	√	√	√	
			*Iris bloudowii*		√				
	*Alismataceae*	*Alisma*	*Alisma orientale*		√	√	√		
			*Alisma nanum*						
	*Boraginaceae*	*Asperugo*	*Asperugo procumbens*						
		*Lappula*	*Lappula myosotis*			√			
			*Lappula semiglabra*						
		*Cynoglossum*	*Cynoglossum viridiflorum*						
		*Myosotis*	*Myosotis alpestris*		√	√		√	√
	*Plantaginaceae*	*Plantago*	*Plantago asiatica*	√		√			√
			*Plantago major*	√		√			
			*Plantago maxima*						
		*Veronica*	*Veronica undulata*			√			
	*Lamiaceae*	*Mentha*	*Mentha canadensis*	√		√			√
		*Lycopus*	*Lycopus europaeus*			√			
		*Scutellaria*	*Scutellaria scordifolia*		√	√			
			*Scutellaria galericulata*			√			
		*Lophanthus*	*Lophanthus chinensis*			√			
		*Stachys*	*Stachys palustris*			√			
		*Prunella*	*Prunella vulgaris*	√		√			
		*Leonurus*	*Leonurus japonicus*		√	√			
	*Euphorbiaceae*	*Euphorbia*	*Euphorbia esula*			√		√	
	*Cannabaceae*	*Cannabis*	*Cannabis sativa*			√		√	√
		*Humulus*	*Humulus lupulus*	√	√	√			
	*Juncaceae*	*Juncus*	*Juncus compressus*			√	√	√	
	*Apocynaceae*	*Cynanchum*	*Cynanchum acutum subsp. sibiricum*	√		√			
	*Hypericaceae*	*Hypericum*	*Hypericum perforatum*			√			
	*Violaceae*	*Viola*	*Viola altaica*						
			*Viola mirabilis*			√			
	*Malvaceae*	*Lavatera*	*Lavatera cashemiriana*		√				
		*Malva*	*Malva verticillata*	√		√			
		*Alcea*	*Althaea rosea*	√	√	√		√	√
		*Althaea*	*Althaea officinalis*	√		√		√	
	*Orchidaceae*	*Platanthera*	*Platanthera minutiflora*						√
		*Spiranthes*	*Spiranthes sinensis*		√	√			
	*Melanthiaceae*	*Veratrum*	*Veratrum nigrum*			√			
	*Polygonaceae*	*Polygonum*	*Polygonum aviculare*	√		√	√		
		*Persicaria*	*Polygonum hydropiper*	√	√	√			
		*Rumex*	*Rumex acetosa*			√			√
			*Rumex crispus*			√			
	*Onagraceae*	*Chamerion*	*Chamerion angustifolium*	√	√	√	√	√	
		*Epilobium*	*Epilobium hirsutum*	√	√	√			
	*Gentianaceae*	*Centaurium*	*Centaurium pulchellum*						
	*Portulacaceae*	*Portulaca*	*Portulaca oleracea*	√		√			
	*Geraniaceae*	*Geranium*	*Geranium wilfordii*		√	√			√
	*Lythraceae*	*Lythrum*	*Lythrum salicaria*	√	√	√		√	
	*Juncaginaceae*	*Triglochin*	*Triglochin palustris*			√			
	*Santalaceae*	*Thesium*	*Thesium chinense*			√			
	*Asparagaceae*	*Asparagus*	*Asparagus officinalis*	√	√	√			
			*Asparagus neglectus*			√			
	*Mazaceae*	*Dodartia*	*Dodartia orientalis*			√			
	*Solanaceae*	*Solanum*	*Solanum dulcamara*						
	*Paeoniaceae*	*Paeonia*	*Paeonia anomala*		√	√			
	*Amaryllidaceae*	*Allium*	*Allium ramosum*	√	√	√			√
	*Typhaceae*	*Sparganium*	*Sparganium stoloniferum*		√	√		√	
	*Plumbaginaceae*	*Limonium*	*Limonium gmelinii*		√	√	√	√	
	*Primulaceae*	*Lysimachia*	*Lysimachia davurica*	√	√	√			

‘√’ indicates an occurrence.

**Table A2:** Distribution of wild vascular plants in rivers.

Species	Bilizik River	Haba River	Burqin River	Crane River	Kayertes River	Kuyertes River	Trunk Stream
*Abies sibirica*				√			
*Acer negundo*				√			
*Achillea millefolium*		√	√	√	√	√	√
*Aconitum carmichaeli*				√	√	√	
*Aconitum smirnovii*			√	√			
*Aegopodium alpestre*		√		√			
*Aeluropus micrantherus*				√	√	√	√
*Aeluropus pungens*		√					
*Agrimonia pilosa*			√	√			
*Agropyron cristatum*		√					
*Agrostis gigantea*		√	√	√	√	√	√
*Agrostis stolonifera*			√				
*Alchemilla pinguis*				√			
*Alisma nanum*				√			√
*Alisma orientale*							√
*Allium ramosum*			√				√
*Alopecurus aequalis*				√			
*Althaea officinalis*							√
*Althaea rosea*				√			
*Alyssum linifolium*				√			
*Angelica sylvestris*		√					
*Apocynum venetum*		√		√			√
*Arctium lappa*		√		√			
*Arctium tomentosum*		√					
*Artemisia annua*				√	√	√	√
*Artemisia lavandulaefolia*				√	√	√	√
*Artemisia scoparia*				√			√
*Artemisia vulgaris*	√	√	√				
*Asparagus neglectus*		√					√
*Asparagus officinalis*							√
*Asperugo procumbens*							√
*Aster altaicus*		√	√				
*Bassia scoparia*		√		√			√
*Berberis iliensis*				√			
*Betula pendula*	√	√	√	√	√	√	
*Bidens tripartita*							√
*Bidens cernua*				√			√
*Bolboschoenus planiculmis*			√	√			√
*Bromus inermis*		√	√	√	√	√	√
*Bromus japonicus*					√	√	
*Calamagrostis epigeios*	√	√		√			√
*Calystegia hederacea*							√
*Cannabis sativa*	√	√	√	√	√	√	√
*Capsella bursapastoris*				√			
*Caragana arborescens*				√			
*Carduus nutans*		√					
*Carex* *altaica*				√			√
*Carum carvi*		√	√	√			√
*Cenolophium denudatum*				√			√
*Centaurium pulchellum*				√			
*Cerastium lithospermifolium*				√			
*Cerastium arvense subsp. strictum*			√				
*Ceratocarpus arenarius*							√
*Chamerion angustifolium*		√		√			√
*Chenopodium acuminatum*							√
*Chenopodium album*				√			
*Chenopodium ficifolium*				√		√	√
*Chloris virgata*						√	
*Cichorium intybus*				√		√	
*Cirsium arvense*							√
*Cirsium arvense var. integrifolium*			√	√		√	√
*Clematis glauca*	√		√				√
*Clematis integrifolia*				√			√
*Clematis intricata*				√			
*Convolvulus arvensis*		√	√	√			√
*Conyza canadensis*		√	√				√
*Corispermum lehmannianum*				√			√
*Cotoneaster melanocarpus*	√		√				√
*Crataegus altaica*	√	√	√	√		√	√
*Cuscuta chinensis*				√			√
*Cynanchum acutum subsp. sibiricum*				√			√
*Cynoglossum viridiflorum*				√			√
*Cyperus fuscus*				√			
*Cyperus michelianus*		√					
*Dactylis glomerata*		√					
*Delphinium eglandulosum*			√	√			
*Dodartia orientalis*				√			√
*Dysphania botrys*							√
*Echinochloa crus-galli*		√		√	√	√	√
*Echinops sphaerocephalus*		√		√			√
*Elaeagnus angustifolia*				√			
*Eleocharis yokoscensis*		√					
*Elymus dahuricus*		√	√	√	√	√	√
*Elymus gmelinii*		√	√				
*Elytrigia repens*		√					
*Ephedra equisetina*		√					
*Epilobium hirsutum*		√					
*Equisetum arvense*		√	√	√	√	√	√
*Equisetum fluviatile*		√	√				
*Equisetum hyemale*	√	√	√	√		√	√
*Equisetum ramosissimum*		√		√			√
*Eragrostis minor*				√			
*Eragrostis pilosa*		√					√
*Erigeron pseudoseravschanicus*				√	√	√	
*Erigeron acris*		√		√			√
*Eryngium planum*				√			√
*Euphorbia esula*							√
*Filipendula ulmaria*			√				
*Fragaria vesca*			√	√			
*Galium odoratum*		√	√			√	
*Galium verum*				√			√
*Galium boreale*		√	√	√			√
*Galium soongoricum*				√			
*Geranium wilfordii*		√	√	√	√	√	√
*Geum aleppicum*		√	√	√			
*Glycyrrhiza uralensis*	√		√	√		√	√
*Gypsophila paniculata*				√			√
*Halimodendron halodendron*	√			√			√
*Heracleum dissectum*				√			√
*Hieracium umbellatum*		√	√		√	√	
*Hordeum bogdanii*		√		√			
*Humulus lupulus*	√	√	√	√			√
*Hypericum perforatum*				√			
*Inula britanica*		√					
*Inula helenium*		√	√	√			√
*Inula japonica*	√	√	√			√	√
*Iris lactea var. chinensis*							√
*Iris bloudowii*							√
*Juncus compressus*		√	√	√			
*Lactuca seriola*				√			√
*Lactuca tatarica*	√	√	√	√			√
*Lappula myosotis*		√		√			√
*Lappula semiglabra*		√					
*Larix gmelini*				√			
*Lathyrus davidii*			√				
*Lathyrus pratensis*	√				√	√	
*Lavatera cashemiriana*			√				
*Leonurus japonicus*				√			
*Lepidium latifolium*			√	√			
*Lepidium chalepense*				√			
*Leymus paboanus*		√					
*Leymus secalinus*	√	√	√				
*Limonium gmelinii*				√			√
*Linum usitatissimum*							√
*Lolium perenne*				√			
*Lonicera caerulea*			√				
*Lonicera tatarica*	√	√		√			
*Lophanthus chinensis*		√					
*Lotus corniculatus*		√	√	√			√
*Lycopus europaeus*				√			√
*Lysimachia davurica*		√	√	√			√
*Lythrum salicaria*		√	√	√			√
*Malus sylvestris*				√			
*Malva verticillata*				√			
*Medicago falcata*	√	√	√	√			√
*Medicago lupulina*			√	√			√
*Medicago sativa*		√	√	√	√	√	√
*Melilotus alba*			√	√			
*Melilotus officinalis*							√
*Melilotus suaveolens*				√			
*Mentha canadensis*		√	√	√			√
*Myosotis alpestris*		√					
*Neotrinia splendens*	√			√		√	√
*Oxybasis glauca*	√	√	√	√	√	√	√
*Paeonia anomala*			√	√			
*Parasenecio hastatus*		√					
*Phleum pratense*		√					
*Phragmites australis*		√	√	√	√	√	√
*Picea obovata*			√	√	√	√	
*Plantago asiatica*	√	√		√	√	√	√
*Plantago major*			√	√			√
*Plantago maxima*				√			
*Platanthera minutiflora*		√					
*Poa annua*	√	√	√	√	√	√	√
*Poa nemoralis*	√	√	√	√			√
*Poa pratensis subsp. angustifolia*	√	√	√				
*Polygonum aviculare*		√		√		√	√
*Polygonum hydropiper*		√		√			√
*Populus alba*	√	√		√			√
*Populus canescens*	√	√					
*Populus euphratica*							√
*Populus laurifolia*	√	√	√	√	√	√	√
*Populus nigra*	√	√		√			√
*Populus pilosa*		√					
*Populus tremula*		√		√			
*Populus × berolinensis var. jrtyschensis*	√	√	√	√			√
*Portulaca oleracea*		√					
*Potentilla anserina*		√	√	√			√
*Potentilla chinensis*				√	√	√	
*Potentilla desertorum*				√			√
*Potentilla fruticosa*			√				
*Potentilla supina*		√		√		√	√
*Prunella vulgaris*		√	√				
*Ranunculus altaicus*				√			
*Rhaponticum repens*				√			√
*Rorippa palustris*				√			
*Rosa acicularis*	√	√	√		√	√	
*Rosa laxa*	√	√	√	√		√	√
*Rubia dolichophylla*				√	√	√	√
*Rubia rezniczenkoana*		√					
*Rubus saxatilis*		√	√		√	√	
*Rubus idaeus*			√				
*Rumex acetosa*	√		√	√	√	√	√
*Rumex crispus*			√				
*Sagina saginoides*				√			
*Salix alba*	√	√	√	√			√
*Salix bulkingensis*			√				
*Salix caspica*	√	√		√		√	√
*Salix cinerea*	√						
*Salix dasyclados*	√		√	√	√	√	√
*Salix pyrolifolia*			√	√			
*Salix nipponica*		√					
*Salsola abrotanoides*							√
*Salsola collina*			√	√			√
*Sanguisorba officinalis*		√					
*Schoenoplectus tabernaemontani*		√		√			√
*Schoenoplectus triqueter*		√					
*Scutellaria galericulata*				√			
*Scutellaria scordifolia*		√	√	√			√
*Senecio argunensis*	√	√	√	√			√
*Seriphidium terraealbae*		√		√			√
*Setaria viridis*		√	√	√	√	√	√
*Sibbaldianthe bifurca*		√			√	√	√
*Silene graminifolia*				√			
*Sium suave*	√	√	√	√			√
*Solanum dulcamara*				√			√
*Solidago virgaurea*				√			
*Sonchus brachyotus*			√	√			√
*Sonchus oleraceus*		√		√			
*Sonchus transcaspicus*		√	√	√			√
*Sophora alopecuroides*	√	√	√	√		√	√
*Sparganium stoloniferum*							√
*Sphaerophysa salsula*				√			√
*Spiraea elegans*				√			
*Spiraea hypericifolia*					√	√	
*Spiranthes sinensis*				√			
*Stachys palustris*		√		√			
*Stellaria media*		√					
*Tamarix chinensis*							√
*Taraxacum mongolicum*	√	√	√	√		√	√
*Thalictrum flavum*	√	√	√	√	√	√	√
*Thalictrum simplex*		√	√				√
*Thesium chinense*		√					
*Thlaspi arvense*							√
*Tragopogon orientalis*							√
*Trifolium fragiferum*							√
*Trifolium hybridum*			√	√			
*Trifolium lupinaster*		√	√	√	√	√	
*Trifolium pratense*		√	√	√	√	√	√
*Trifolium repens*	√	√	√	√			√
*Triglochin palustris*				√			
*Tussilago farfara*		√		√			
*Ulmus pumila*				√			
*Vaccinium myrtillus*				√			
*Veratrum nigrum*		√					
*Veronica undulata*				√			
*Viburnum opulus*	√	√					√
*Vicatia atrosanguinea*				√			
*Vicia cracca*		√	√	√			√
*Vicia lilacina*		√					
*Vicia pseudorobus*		√					
*Vicia sativa subsp. nigra*		√	√	√	√	√	√
*Vicia sepium*		√	√	√			
*Viola altaica*				√			
*Viola mirabilis*		√	√	√			
*Xanthium sibiricum*	√			√		√	√
Total	43	123	94	167	37	54	131

‘√’ indicates an occurrence.
